# Releasing 8.0 wt.% H_2_ from the LiBH_4_−2LiNH_2_ Composite within 5 Min under Light Illumination

**DOI:** 10.1002/advs.202501140

**Published:** 2025-06-04

**Authors:** Haoyang Yu, Zibo Cheng, Hong Wen, Han Wang, Qijun Pei, Yeqin Guan, Hujun Cao, Ping Chen

**Affiliations:** ^1^ Dalian Institute of Chemical Physics Chinese Academy of Sciences Dalian 116023 China; ^2^ Center of Materials and Optoelectronics Engineering University of Chinese Academy of Sciences Beijing 100049 China; ^3^ School of Chemistry and Chemical Engineering Henan University of Technology Zhengzhou 450001 China; ^4^ State Key Laboratory of Catalysis Dalian 116023 China

**Keywords:** LiBH_4_−2LiNH_2_, non‐thermal effect, photo‐driven dehydrogenation, photothermal effect, reactive hydride composite

## Abstract

The absence of safe and efficient hydrogen storage technologies is the major bottleneck for widespread applications of hydrogen energy. Reactive hydride composites with high gravimetric and volumetric hydrogen densities are ideal hydrogen storage materials. However, their traditional dehydrogenation processes normally involving electric‐thermal‐chemical energy conversion require high operating temperatures and substantial energy inputs to heat the reactor and oven. In this study, using LiBH_4_−2LiNH_2_ as a model system, that rapid dehydrogenation via a photo‐thermal‐chemical and/or photo‐chemical energy conversion initiated by direct light irradiation is demonstrated and can be fulfilled in the presence of a catalyst and a photothermal agent. The experimental results revealed that the non‐thermal effect of UV light plays a critical role in reducing the desorption temperature and enhancing the dehydrogenation kinetics. The collective photothermal and non‐thermal effects drove over 8.0 wt.% hydrogen desorption from LiBH_4_−2LiNH_2_ within 5 min, which is ≈60 times faster than the thermal dehydrogenation process at the same temperature.

## Graphical abstract

The N−H bond in the [NH_2_]^−^ ion group is significantly weakened by the non‐thermal effect of UV light, leading to a 60‐fold increase in dehydrogenation rate at the same temperature. As a result, over 8.0 wt.% H_2_ is released within 5 min.

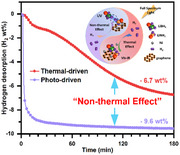

## 1 Introduction

Hydrogen, a versatile energy carrier, plays a pivotal role in clean energy storage, power generation, and the chemical industry.^[^ ^1^ ^]^ While considerable efforts have been devoted to developing safe and efficient hydrogen storage technologies, significant challenges remain, limiting the step into the hydrogen economy.^[^ ^2^ ^]^ Among various approaches, the reactive hydride composites (RHCs) have emerged as highly promising solid‐state hydrogen storage materials, following the groundbreaking discovery by Chen et al. that the LiNH_2_−2LiH system can reversibly store 10.4 wt.% hydrogen.^[^ ^3^ ^]^ This performance was attributed to the synergistic interaction between hydridic (H^δ−^) atoms in LiH and protic (H^δ+^) atoms in [NH_2_]^−^ groups, which drives hydrogen release.^[^ ^4^ ^]^ Subsequently, a range of RHCs, including LiBH_4_−MgH_2_, MgH_2_−LiNH_2_, and LiBH_4_−2LiNH_2_, have been extensively investigated.^[^ ^5^ ^]^ Notably, the LiBH_4_−2LiNH_2_ (hereafter referred to as LiBNH) was first synthesized via ball‐milling by Pinkerton et al. in 2005 (Equation (1)), stands out for its convenience as well as superior dehydrogenation performance compared to its individual components under identical conditions, releasing over 10.0 wt.% of hydrogen within a temperature range of 250–350 °C (Equation (2)).^[^ ^6^ ^]^

(1) $$\mathrm{LiB}H_{4}+2\mathrm{LiN}H_{2}\to \left[Li_{3}BN_{2}H_{8}\right]$$

(2) $$\left[Li_{3}BN_{2}H_{8}\right]\to Li_{3}BN_{2}+4H_{2}$$

This high hydrogen storage capacity positions LiBNH as a potential candidate for practical applications, provided that its dehydrogenation temperature can be significantly reduced. Incorporating highly efficient catalysts, such as transition metals and their derivatives (e.g., chlorides and oxides), has proven to be a viable strategy to achieve this goal.^[^ ^7^ ^]^ Pinkerton et al. reported that the peak dehydrogenation temperature of 11 wt.% NiCl_2_‐doped LiBNH is dropped by ≈112 °C compared to the unmodified one (ca. 340 °C).^[^ ^8^ ^]^ Nanosized Ni@C composite and flower‐like nickel oxide (NiO) have demonstrated the capability to release ≈10.0 wt.% H_2_ within 21 min at temperatures above 220 °C.^[^ ^7^, ^9^ ^]^ Mechanism analyses revealed that during dehydrogenation, NiO is reduced to metallic Ni, which acts as the catalytically active species, thereby reducing the reaction barriers for dehydrogenation.

Recently, various external fields, such as ultrasonic, plasma, electric field, microwave, and light field, have been employed or integrated with conventional thermal methods to drive H_2_ release.^[^ ^10^ ^]^ Light‐driven dehydrogenation has attracted significant attention due to its non‐polluting, renewable, and economical nature. Sun et al. used the heat generated by the plasmonic effect of Au nanoparticles to drive the dehydrogenation of NaAlH_4_, MgH_2_ as well as LiH.^[^ ^10f^ ^]^ Cheng et al. discovered that the LiH surface can reversibly store H_2_ under UV illumination, indicating an interaction between UV light and hydrides.^[^ ^10k^ ^]^ Zhang et al. revealed that by coupling the photothermal effect of light agents (i.e., MXene and Cu) with the catalytic effect of thermochemical catalysts (i.e., Ti/TiH_x_), the hydrogen storage performance of MgH_2_ induced by photothermal effects is significantly improved.^[^ ^10j^ ^]^

Theoretically, using solar energy to drive hydrogen release is significantly useful in some special scenarios where other energies are unavailable, such as wilderness, desert areas, outer space etc. Motivated by this, we incorporated light into the LiBH_4_−2LiNH_2_ (LiBNH) composite, utilizing nickel oxide as a catalyst and graphene as a photothermal agent. The dehydrogenation kinetics of LiBNH were significantly enhanced through the combination of non‐thermal and photothermal effects under full‐spectrum light. At an illumination intensity of 1.94 W cm^−2^ (the temperature of the sample surface is ≈180 °C), over 8.0 wt.% H_2_ was released within 5 min and ultimately achieved a yield of ca. 9.6 wt.% H_2_. Experimental and theoretical analyses revealed that, in addition to the thermal effect of visible light on the material, the interaction of UV light with the [NH_2_]^−^ group, which weakens the N−H bond at low temperatures, plays a vital role in the rapid dehydrogenation of the LiBNH system. This study represents an inaugural report on the synergistic effects of non‐thermal and photothermal in enhancing the hydrogen storage performance of reactive hydride composites.

## 2 Results and Discussion

### 2.1 Photo‐Driven Dehydrogenation of LiBNH

**Figure 1:**
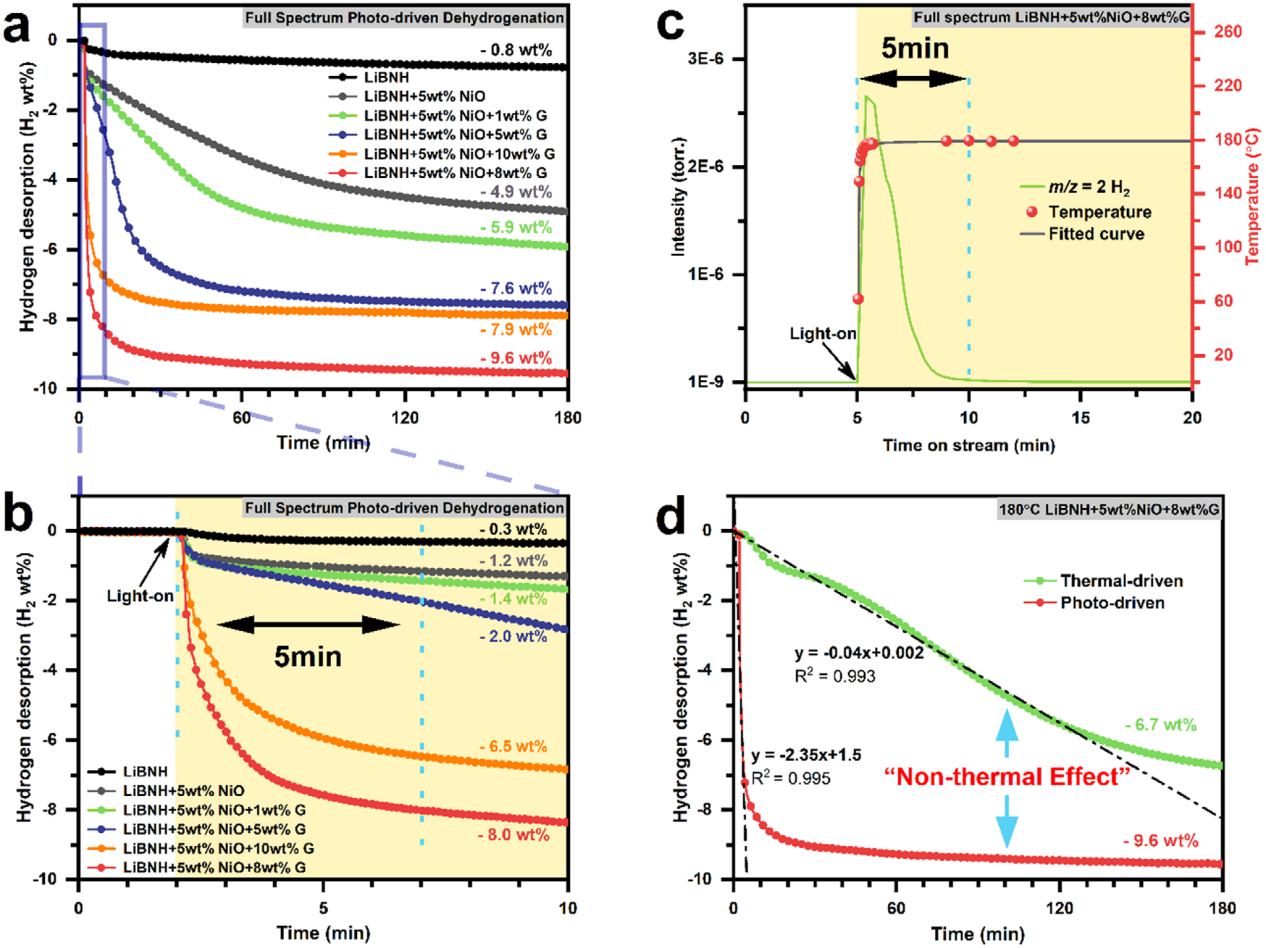
a,b) Photo‐driven volumetric dehydrogenation of as‐prepared samples within 180 and 10 min, respectively, under full‐spectrum illumination of 1.94 W cm^−2^. c) Photo‐driven dehydrogenation of LiBNH+5wt.%NiO+8wt.%G in the mass spectrometry with fitted real‐time temperature. d) Thermal and photo‐driven volumetric dehydrogenation comparison of LiBNH+5wt.%NiO+8wt.%G at isothermal 180 °C.

LiBNH and LiBNH+5wt.%NiO+*x*wt.%G (G: graphene) composites have been synthesized by ball‐milling the related substrates (Experimental section). The additional amount of NiO is 5 wt.% based on Wu's results.^[^ ^7e^ ^]^ The pristine LiBNH itself does not exhibit noticeable light absorption in the range of 270–900 nm. However, the LiBNH+5wt.%NiO+8wt.%G composite exhibits a significant absorption throughout the entire range (Figure S1, Supporting Information). Photo‐driven dehydrogenation of all the as‐prepared samples at an irradiation intensity of 1.94 W cm^−2^ for 3 h is summarized in **Figure** **1**a. Dehydrogenation kinetics and capacities are significantly improved in all the graphene‐added samples. The LiBNH+5wt.%NiO+8 wt.%G shows the best dehydrogenation properties, releasing 9.6 wt.% H_2_ in 3 h, while only 4.9 wt.% H_2_ is liberated in the LiBNH+5wt.%NiO under the same conditions. As the graphene doping amount is further increased, the dehydrogenation capacity decreases rather than further increases, as seen in LiBNH+5wt.%NiO+10wt.%G. Figure 1b is a close‐up of Figure 1a within the first 10 min. Notably, the LiBNH+5wt.%NiO+8wt.%G exhibits exceptional performance, releasing over 5.8 and 8.0 wt.% H_2_ within 1 and 5 min, respectively, accounting for 65% and 85% of the total available hydrogen, which is ≈30‐fold greater than that of the pristine LiBNH (0.3 wt.%, 5 min). To maximize the differences brought by photo‐driven and thermal‐driven dehydrogenation within the same catalyst, the LiBNH+5wt.%NiO+8wt.%G is selected for photo‐driven dehydrogenation under the same illumination with fitted real‐time temperature (Figure 1c,d). Similar to volumetric dehydrogenation, hydrogen release begins immediately upon light irradiation, and the dehydrogenation process is almost finished within 5 min. The in situ real‐time temperature is measured through the infrared detector, as shown in Figures S2 and S3 (Supporting Information), indicating that the surface temperature of the LiBNH+5wt.%NiO+8wt.%G is raised to 180 °C within only 45 s of light irradiation and remains constant thereafter. The thermal temperature‐programmed dehydrogenation (Figure S4a, Supporting Information) shows that the initial dehydrogenation temperature is ≈125 °C, confirming that photothermal effects drive H_2_ release in these composites under full‐spectrum illumination. The photo‐driven dehydrogenation rate is ≈60 times higher than the thermal‐driven process at the first 2 h, which confirms that photo‐driven dehydrogenation has a different reaction mechanism and offers benefits over thermal dehydrogenation (Figure 1d).

### 2.2 Non‐Thermal Effect and Structural Transformation of LiBNH under Light Illumination

**Figure 2:**
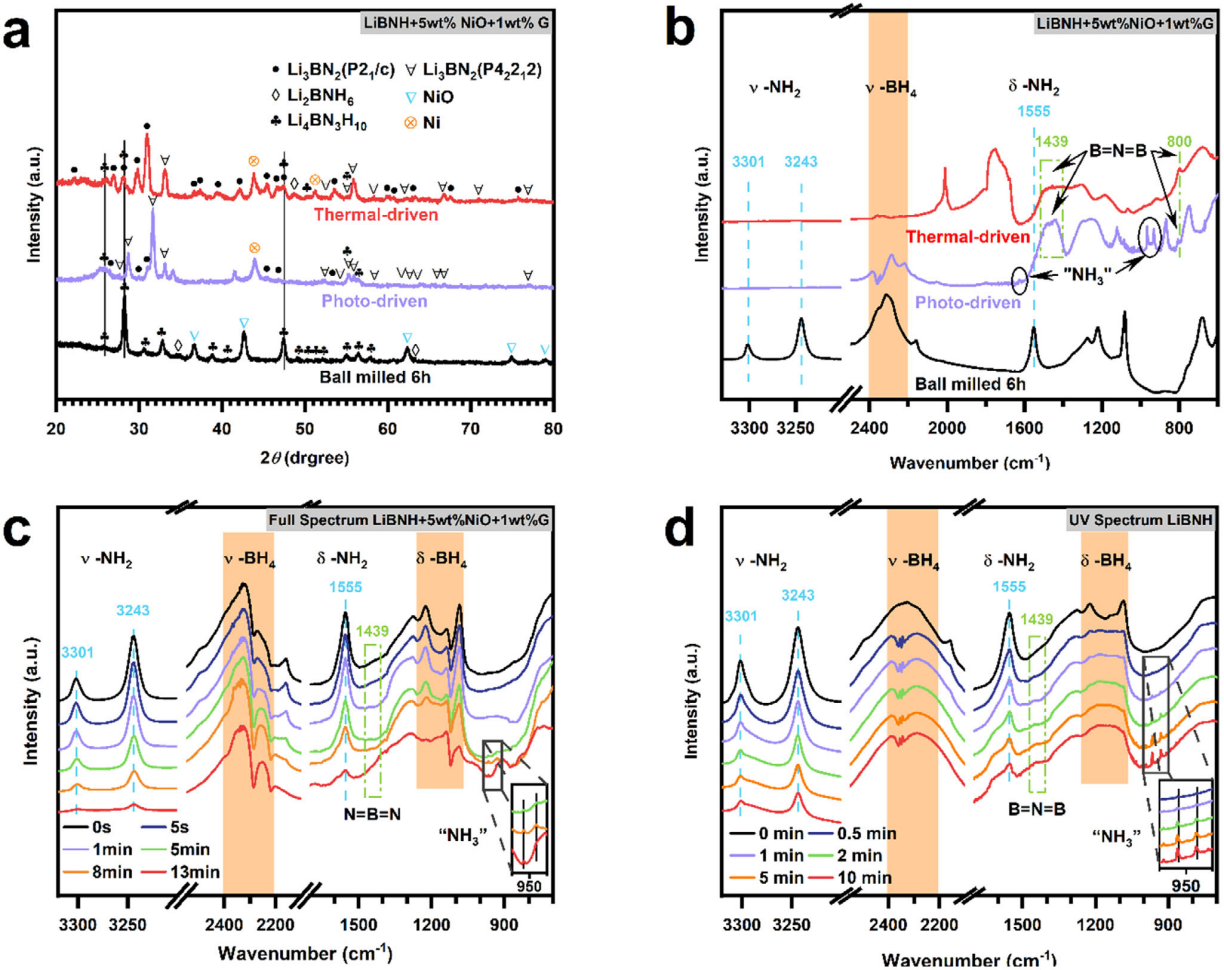
a) XRD patterns and b) FT‐IR spectra of the as‐prepared LiBNH+5wt.%NiO+1wt.%G before and after dehydrogenation under thermal and photo‐driven processes. c,d) Semi‐in situ FT‐IR spectra of the LiBNH+5wt.%NiO+1wt.%G under full‐spectrum (0.98 W cm^−2^) and UV illumination, respectively.

To understand the influence of the non‐thermal effect of light in dehydrogenation, the as‐synthesized and dehydrogenated LiBNH+5wt.%NiO+1wt.%G during thermal and photo‐driven dehydrogenation processes have been collected for XRD and FT‐IR measurements. The sample with 1 wt.%G is chosen for measurements due to its minimal light‐absorption capacity on the infrared test. **Figure** **2**a shows that peaks of Li_4_BN_3_H_10_, Li_2_BNH_6_, and NiO exist in the as‐prepared sample. Almost all the Li_4_BN_3_H_10_ and Li_2_BNH_6_ in the sample are transformed into Li_3_BN_2_ after dehydrogenation, and two different structures of Li_3_BN_2_ can be identified, including the room‐temperature (P4_2_2_1_2) and the high‐temperature (P2_1_/c) structures.^[^ ^11^ ^]^ Most of the room‐temperature structure is detected in the photo‐driven dehydrogenated sample. However, high‐temperature structure is the primary one in the thermal‐driven dehydrogenated sample. Additionally, nickel oxide is reduced to metallic nickel during both the dehydrogenation processes. FT‐IR results clearly show that the characteristic stretch peaks of N─H bonds at 3301 and 3243 cm^−1^, corresponding to Li_4_BN_3_H_10_ and Li_2_BNH_6_, are observed in Figure 2b.^[^ ^12^ ^]^ After thermal‐driven dehydrogenation to 500 °C, the N−H and B−H vibrations at 3301, 3243, and 1555 cm^−1^ completely vanished, along with the appearances of N ═ B ═ N bonds centered at 1440 and 800 cm^−1^.^[^ ^13^ ^]^ Similar results are observed in the photo‐driven dehydrogenated sample under full‐spectrum illumination for 5 min. Nevertheless, it is observed that the peaks at 1626, 967, and 931 cm^−1^ corresponding to adsorbed ammonia (evidenced later), are observed on the sample surface (black circle), which is highly possible due to that LiBH_4_ species absorb ammonia forming LiBH_4_•xNH_3_.^[^ ^14^ ^]^ This sign means different dehydrogenation driving forces lead to disparate reaction processes.

To further understand the photo‐driven dehydrogenation mechanism, semi‐in situ infrared spectroscopy was used to elucidate the dehydrogenation process under full‐spectrum illumination at a lower intensity (0.98 W cm^−2^) and confirm the details of peak changes. Figure 2c shows that the intensity of N−H bonds at 3301, 3243, and 1555 cm^−1^ gradually disappears or rapidly weakens with the prolongation of light irradiation over 13 min. The same phenomenon happens with B−H bonds, where the intensity of the B−H vibration band decreases with increased irradiation time. The interaction between N−H and UV illumination occurs at ambient temperature, and the intensity of N−H bonds at 3301, 3243, and 1555 cm^−1^ is visibly decreased. The vibrations of the B−H bonds are also changed under UV illumination. Besides, the gradual appearance of B−N vibrations centered at 1440 cm^−1^ is observed, indicating that UV light triggers the dehydrogenation process. The most interesting finding is that the N−H vibration signals of absorbed NH_3_ appear at 967 and 931 cm^−1^ during the UV irradiation process, which cannot be observed in the thermal‐driven dehydrogenation process (Figure 2b). Based on the evidence mentioned above, it is reasonable to conjecture that under UV illumination, the activation of N−H and B−H bonds leads to the formation of an intermediate state for absorptive ammonia at room temperature. This intermediate may further interact with the B−H group, facilitating H_2_ release, which also explains the change in the vibrations of the B−H bonds under UV irradiation. However, the release of gas ammonia is only detectable at temperatures above 100 °C during the thermal‐driven reaction (Figure S4a, Supporting Information), suggesting that the non‐thermal effect of UV light may be the fundamental reason for the appearance of absorbed ammonia vibrations at room temperature.

**Figure 3:**
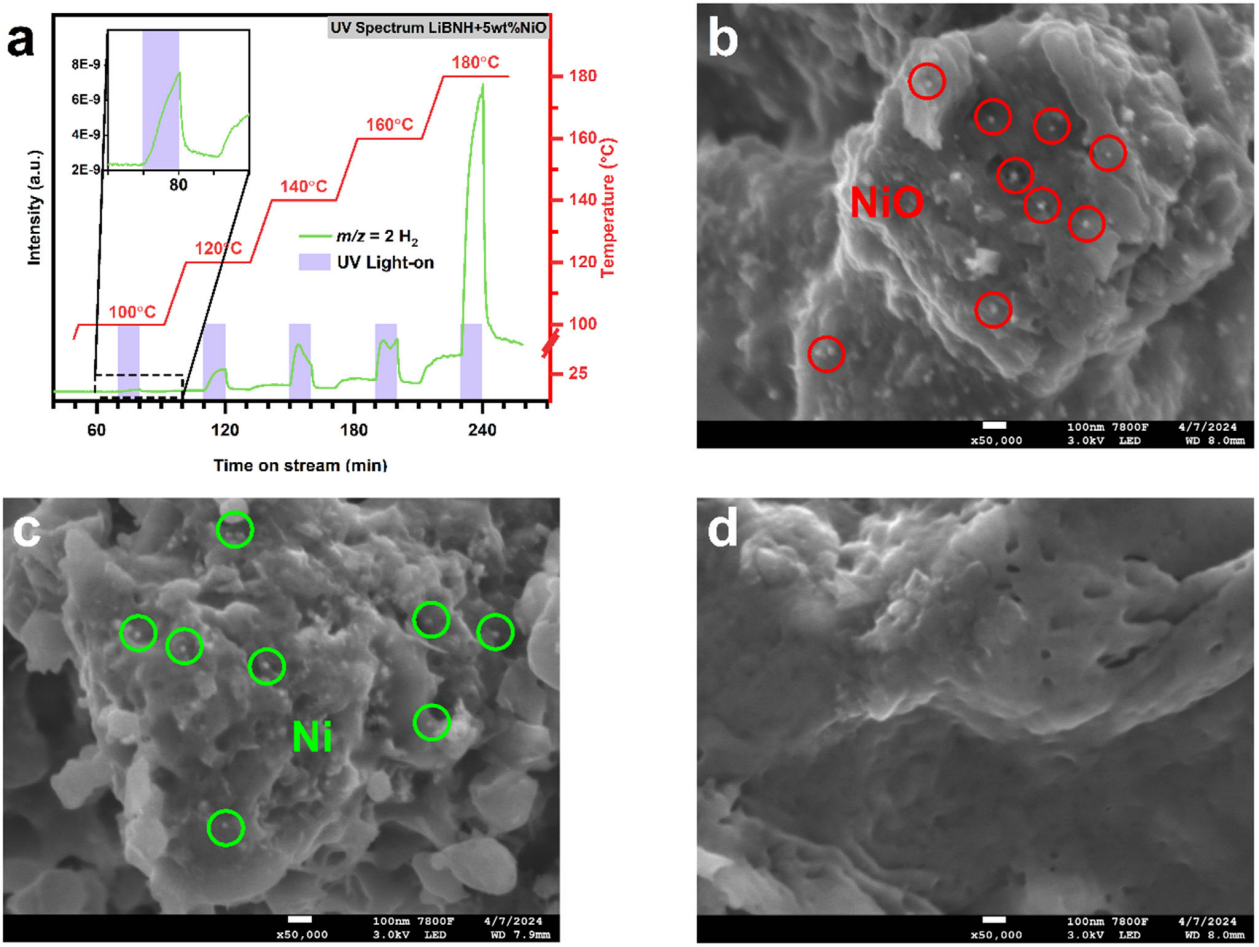
a) Photo‐driven dehydrogenation under periodicity UV illumination in the mass spectrometry at different isothermal temperatures. SEM images of LiBNH+5wt.%NiO+8wt.%G b) as–prepared, c) dehydrogenated by full‐spectrum light (1.94 W cm^−2^, 180 °C) and d) thermal dehydrogenated to 180 °C.

To further elucidate the non‐thermal effect, dehydrogenation of the LiBNH+5wt.%NiO+8wt.%G by combining UV irradiation and isothermal conditions was conducted. **Figure** **3**a shows that when LiBNH+5wt.%NiO+8wt.%G is heated below 100 °C, there is rarely hydrogen production. However, when applying UV light at this temperature, the intensity of H_2_ increases more than 3 times (inset figure). In the whole measured temperature range of 100 to 180 °C, enhanced hydrogen release ability is consistently observed with each intermittent UV illumination, and there is a gradual enhancement in the extent of dehydrogenation under continuous UV illumination as the temperature increases. After removing the UV illumination, the dehydrogenation rate of LiBNH+5wt.%NiO+8wt.%G quickly drops. Notably, when the temperature reaches 180 °C, there is a marked increase in the dehydrogenation rate, resulting in substantial H_2_ production, as also evidenced in Figure 1. which suggests that the non‐thermal effect of UV illumination on the dehydrogenation of LiBNH+5wt.%NiO+8wt.%G is significant, and once UV illumination is removed, the dehydrogenation signal in MS is either halted or significantly reduced under thermal‐driven conditions. It is speculated that UV light may not have enough energy to break all the N−H and B−H bonds to release hydrogen, so it requires assistance from thermal energy to jointly release hydrogen. The dehydrogenation rate of LiBNH+5wt.%NiO+8wt.%G under UV irradiation at 140 °C is even higher than that observed at 180 °C without UV irradiation. These observations further confirm the presence of the non‐thermal effect of UV irradiation, suggesting that when combined with appropriate temperature, UV illumination can effectively enhance the kinetics of the dehydrogenation process. This also explains the rapid dehydrogenation under full‐spectrum irradiation, likely due to the synergistic effect of the non‐thermal effect of UV irradiation and the photothermal effects of infrared and visible light, which promotes the release of hydrogen.

The morphological characteristics of LiBNH+5wt.%NiO+8wt.%G at different stages have been examined. As shown in Figure 3b, before dehydrogenation, tiny NiO nanoparticles are uniformly distributed on the surface of the LiBNH+5wt.%NiO+8wt.%G (red circle). After photo‐driven dehydrogenation (Figure 3c), the reduced Ni nanoparticles, which are also shown in Figure 2a, are clearly visible and relatively evenly distributed (green circle). The photo‐driven dehydrogenated sample exhibits minimal morphological changes compared to the as‐synthesized one. In contrast, after thermal dehydrogenation, the entire structure of the material collapses, rendering the catalyst nearly invisible, indicating a tendency toward melting, consistent with previous reports.^[^ ^11a^ ^]^ This is further supported by the observations in the low‐magnification images (Figure S5, Supporting Information). Additionally, the XRD patterns in Figure 2a provide evidence that the room‐temperature structure of Li_3_BN_2_ (P4_2_2_1_2) predominates in the photo‐driven dehydrogenated sample, whereas the high‐temperature structure of Li_3_BN_2_ (P2_1_/c) is the major component in the thermal‐driven dehydrogenated sample. These observations suggest that the non‐thermal effect associated with the light field may inhibit crystal growth, melting, and phase transformation during the reaction, which are critical factors influencing the reaction process.

**Figure 4:**
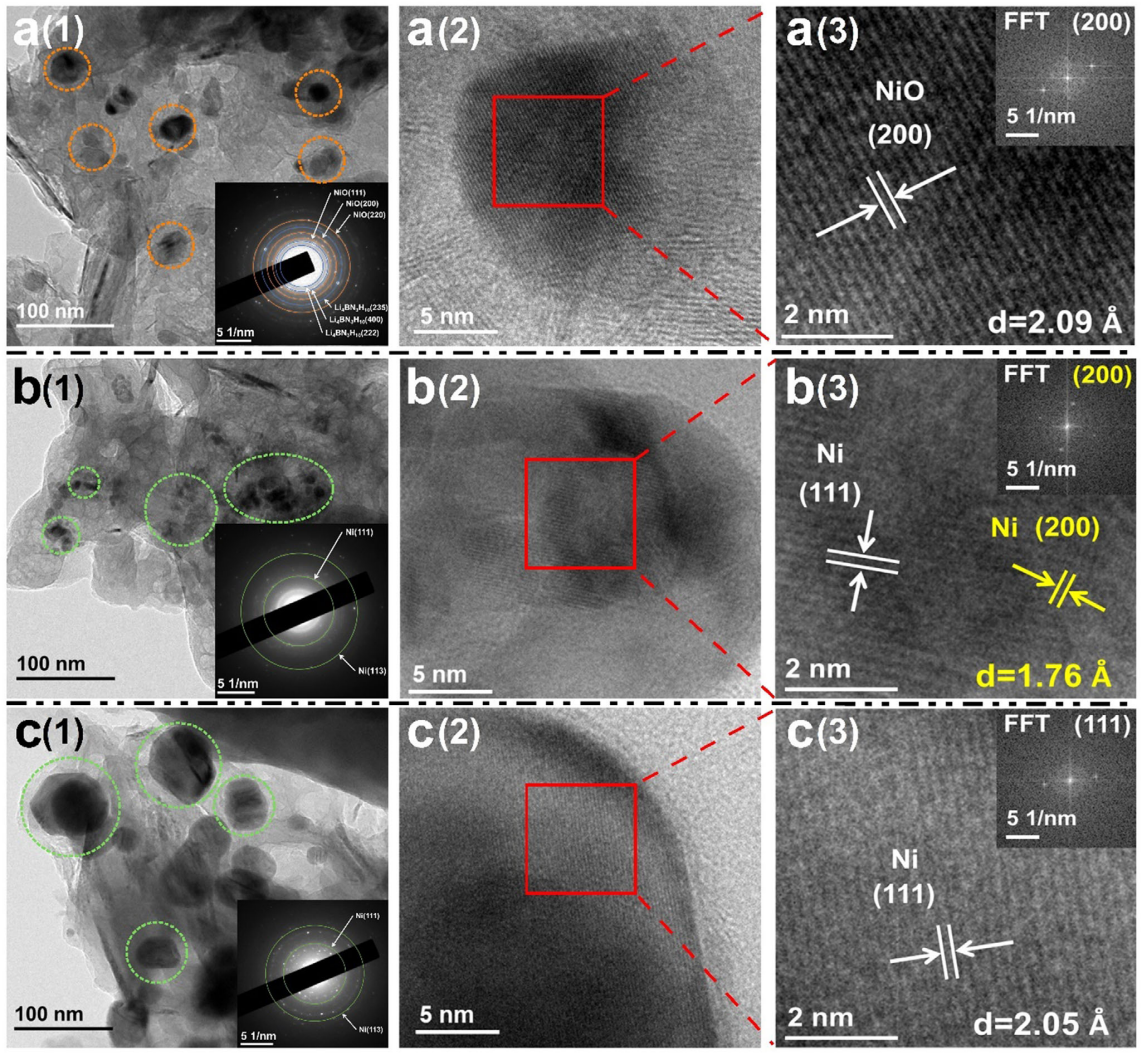
TEM (1), HR‐TEM (2) and the measured typical lattice spacing (3) images of a) as‐prepared, b) photo‐driven and c) thermal‐driven dehydrogenated LiBNH+5wt.%NiO+8wt.%G samples, respectively.

Transmission electron microscopy (TEM) was used to further investigate the microscopic features (**Figure** **4**). As shown in Figure 4a (1), the catalysts are uniformly dispersed within the as‐prepared sample. During the photo‐driven dehydrogenation process (Figure 4b (1)), the growth of crystalline grains is significantly less pronounced compared to that in the thermal‐driven process (Figure 4c (1)). Additionally, HR‐TEM images of the as‐prepared LiBNH+5wt.%NiO+8wt.%G sample (Figure 4a (3)) reveal NiO nanocrystals with the (200) planes as the main exposed facets, which is consistent with the XRD results (Figure 2a). In Figure 4b (3), the white and yellow arrows indicate lattice spacings of 2.05 and 1.76 Å, respectively, belonging to the (111) and (200) crystal planes of metallic Ni in the photo‐driven dehydrogenated sample. In the thermal‐driven dehydrogenated sample (Figure 4c (3)), the white arrow point to lattice fringe of 2.05 Å attributing to the (111) crystal plane of metallic Ni rather than NiO.

### 2.3 Photo‐Driven Dehydrogenation Mechanism of LiBNH

**Figure 5:**
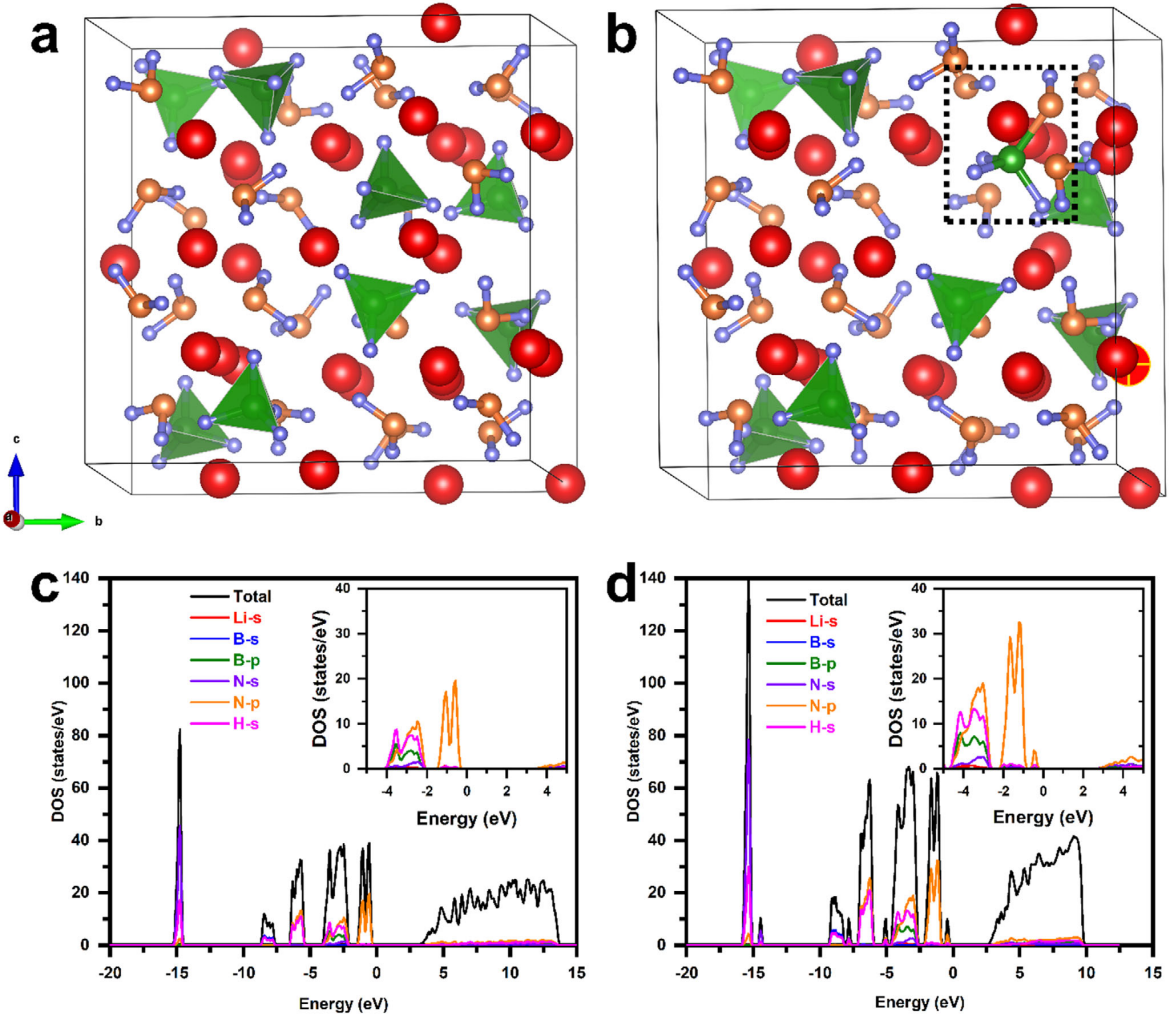
a) The optimized crystal structure of Li_4_BN_3_H_10_ in a ball‐stick view containing [BH_4_]^−^ tetrahedral units (in green and violet) and [NH_2_]^−^ units (in orange and violet), Li^+^ ions are shown as red spheres. b) The optimized crystal structure of Li_4_BN_3_H_10_ with one H_2_ molecule desorption. c) Density of states (DOS) of the Li_4_BN_3_H_10_, and d) Density of states (DOS) of the Li_4_BN_3_H_10_ after removing one H_2_ molecule.

Since LiBNH is a mixture of Li_2_BNH_6_ and Li_4_BN_3_H_10_, as shown in Figure 2a, we constructed and optimized these two crystal structures (**Figure** **5**; Figures S6, S8, and S9, Supporting Information) to investigate the light‐to‐structure interactions. First, we calculated the form energy of one H_2_ molecule in the Li_4_BN_3_H_10_ crystal structure based on the interaction between positive hydrogen from the [NH_2_]^−^ ion group and negative hydrogen from the [BH_4_]^−^ ion group (Figure 5b). The formation energy is 1.24 eV, and some B−H bonds are lengthened from 1.23 to 1.27 Å due to the lattice changes, while Li−N−B bonds form simultaneously with the electron transition (Tables S1 and S3, Supporting Information). Furthermore, the density of states (DOS) of these structures, as shown in Figure 4c,d, reveals the appearance of a hybridized bandgap and a shift in the Fermi energy levels to the right side, indicating the non‐thermal effect of light. Additionally, the formation of ammonia results from the activation of [NH_2_]^−^ by light, as indicated by the dotted box in Figure S6a (Supporting Information). There is a tendency for the hydrogen from the [BH_4_]^−^ ion group to combine with the [NH_2_]^−^ ion group to form an unstable intermediate state, NH_3_, due to the molecular thermal motion or bond activation. This process has a formation energy of 2.71 eV. Moreover, the formation of absorptive ammonia arises from the activation of the [NH_2_]^−^ ion group by light, as shown in Figure S6 (Supporting Information) (dashed box). The releasing gas ammonia has a calculated desorption energy of 0.8 eV, and the formation energy of the total structure is up to 3.51 eV (Figure S6, Supporting Information). Notably, the bandgap in the unstable state, as shown in Figure S6c,d (Supporting Information), is also decreased, which may explain why ammonia can be detected by illuminating LiBNH with UV light at room temperature. It is reasonable to speculate that the UV light has an activating effect on the [NH_2_]^−^ groups, in meanwhile, the [BH_4_]^−^ groups are destabilized, leading to the elongation of B−H bonds during the formation of the ammonia intermediate (Tables S2 and S3, Supporting Information). The above‐mentioned semi‐in situ FT‐IR profile confirms these responses (Figure 2d). Moreover, ammonia emission is detectable during the dehydrogenation processes by thermal and photo‐driven methods, as shown in Figure S7 (Supporting Information), and light illumination can effectively suppress the evolution of NH_3_ by‐products during the dehydrogenation process (Figure S7b, Supporting Information). Generally, under these circumstances, it can be proven that the dehydrogenation process in the Li_4_BN_3_H_10_ lattice has a stronger tendency and a lower forming energy, which explains the excellent photo‐driven dehydrogenation kinetic. Despite the formation of absorptive ammonia intermediate, the higher forming energy blocks this by‐production in some extent, thus increasing the purity of the released hydrogen compared to the thermaldehydrogenation process. Similarly, the respective forming energy and the DOS of the Li_2_BNH_6_ lattice are also obtained, as shown in Figures S8 and S9 (Supporting Information). Although the hot electrons generated by the localized surface plasmon resonance (LSPR) effect of Ni may contribute additional photothermal energy to LiBNH, it appears to be incorporated into the overall thermal effect in this work.^[^ ^15^ ^]^ Therefore, the activation of N−H and B−H bonds by UV and the synergistic effect of the photothermal effect may be the key reasons for the rapid hydrogen release in the LiBNH system under mild conditions.

## 3 Conclusion

With the addition of 8 wt.% graphene (G), the temperature of the LiBNH+5wt.%NiO+8wt.%G composite rapidly increased to 180 °C within 45 s and maintained this temperature throughout the subsequent dehydrogenation process under full‐spectrum illumination of 1.94 W cm^−2^, ultimately facilitated the release of 9.6 wt.% H_2_ within 3 h. Under full‐spectrum illumination, the LiBNH+5wt.%NiO+8wt.%G composite released over 8.0 wt.% hydrogen within just 5 min, with the dehydrogenation rate increased by ≈60 times compared to the thermal dehydrogenation process at the same temperature of 180 °C. Mechanism analysis indicated that the enhancement in dehydrogenation kinetics primarily stems from UV illumination, which selectively destabilizes the N−H and B−H bonds within the LiBNH system via a non‐thermal effect. This non‐thermal effect was synergistically enhanced by the photothermal effect, promoting dehydrogenation under mild conditions. The exclusive use of light as an energy source, coupled with the rapid dehydrogenation kinetics, suggests a promising strategy for the practical application of the LiBNH system in some unique scenarios.

## 4 Experimental Section

### Materials Synthesis

Commercially available lithium amide (>95%, Alfa Aesar), lithium borohydride (>95%, Innochem), and nickel oxide (30 nm, 99.5%, Macklin) were purchased and used as received. The LiBNH composites with/without nickel oxide and graphene were prepared by ball milling the corresponding chemicals on a planetary ball mill (PM−400, Retsch, Germany) at 200 rpm for 6 h. The ball‐to‐sample weight ratio was ≈60:1. All sample preparation, storage, and loading were performed within a glove box (MBRAUN, Germany) filled with pure argon to prevent contamination by moisture and oxygen (H_2_O<0.1 ppm; O_2_<0.1 ppm).

### Performance Evaluation

The hydrogen desorption behavior was qualitatively measured on a homebuilt temperature‐programmed‐desorption (TPD) system coupled with an online mass spectrometer (MS, Hidden, England). The sample (ca. 6 mg) with quartz wool was loaded into a stainless‐steel tube reactor, which was then connected to the TPD system. The temperature was raised from ambient temperature to 400 °C with a ramping rate of 2 °C min^−1^. A constant flow of 30 mL min^−1^ pure argon gas as the carrier gas was maintained during heating. Kinetic hydrogen desorption and volumetric desorption measurements were conducted using a commercial Sieverts‐type high‐pressure gas volume measuring device (PCT, HPSA–auto, China). Approximately 40 mg of the sample was loaded for each test, with a thermocouple inserted, and the hydrogen storage capacity was calculated using the modified Benedict–Webb–Rubin (MBWR) equation of state. For non‐isothermal testing, the sample was gradually heated to 500 °C at a rate of 3 °C min^−1^ for desorption (starting from vacuum ca. 0.001 bar). The temperature and pressure inside the reactor were automatically monitored and recorded. In the isothermal experiment (Figure 1d), the sample was rapidly heated to the desired temperature and held for 10 min to stabilize the temperature before measurement, and then maintained the conditions for the entire period of measurement. Thermogravimetric analyses and differential scanning calorimetry (TG−DSC, German) were performed on a Netzsch STA 449F5 unit. Approximately 5–6 mg of the sample was placed in an aluminum oxide crucible. The temperature was increased from ambient temperature to 300 °C at a heating rate of 2 °C min^−1^ under argon with a carrier flow rate of 100 mL min^−1^.

### Spectrophotometric Determination of Ammonia (indophenol blue colorimetry)

Ammonia in the tail gas was first absorbed by a dilute sulfuric acid solution. Subsequently, 2 mL of the ammonia‐absorbing solution was added to a test tube, followed by sequential addition of 2 mL of 5 wt.% sodium salicylate, 1 mL of 3.5–5 m sodium hypochlorite, and 0.2 mL of 1 wt.% sodium nitroferricyanide. The resulting mixture was incubated in dark at room temperature for 1 h. Deionized water was used as the baseline, and the ammonia−absorbing solution served as the blank control. Absorbance measurements were performed using a UV–vis spectrophotometer (JASCO–750, Japan) in the wavelength range of 500–800 nm, with the characteristic absorption peak for indophenol blue observed at ≈655 nm.

### Characterization

X−ray diffraction (XRD) patterns were obtained using an X'Pert^3^ Materials Research Diffractometer Malvern Panalytical (Netherlands) with Cu Kα radiation (λ = 0.15406 nm, 40 kV, 40 mA) to identify the phase structure of the samples. The XRD data were collected in the 2θ range of 5–90° with a step increment of ca. 0.016° at ambient temperature. The powder samples were sealed in a homemade holder covered with polyimide film to protect against air and moisture during both sample transfer and scanning. Fourier transform infrared (FT–IR, Bruker, German) spectra were obtained with a Tensor II unit. Potassium bromide powder (KBr) was used as a background, and the tests of the as–prepared samples were then executed. All spectra were obtained by averaging 32 scans in a diffuse reflection mode with a resolution of 4 cm^−1^. Scanning electron microscope (SEM, JSM−7800F, Japan) and transmission electron microscope (TEM, JEM–2100, Japan) were employed to examine the morphology of the dehydrogenated products. Samples were quickly transferred from the Argon atmosphere to the facilities with an air exposure time of less than 30 s.

### Characterization—Photo‐Driven Dehydrogenation Measurement

Photo‐driven hydrogen release experiments were conducted in a custom‐made steel reactor with a CaF_2_ window (>95% transmittance in 200–8000 nm) at the top, and the full spectrum light was emitted by a Xenon lamp (CEL–PF300–T8, Beijing Aulight, China) with a total reflector (Figure S10, Supporting Information) or a UV cut‐off reflector (200–400 nm). During all the quantitative dehydrogenation measurements, the light intensity was controlled at 1.94 W cm^−2^, which was measured by a light power meter (PL–MW2000, Perfect light, China). In the qualitative dehydrogenation processes different light intensities of full spectrum and UV‐light were used. Typically, 4 mg tableted sample was loaded in the reactor for the experiment.

## Conflict of Interest

The authors declare no conflict of interest.

## Supporting information



Supporting Information

## Data Availability

The data that support the findings of this study are available from the corresponding author upon reasonable request.
