# Supplementary material for: Releasing 8.0 wt.% H2 from the LiBH4−2LiNH2 Composite within 5 Min under Light Illumination
Source: Adv Sci (Weinh). 2025 Jun 4;12(32):e01140. doi: 10.1002/advs.202501140 (PMC12407394; doi:10.1002/advs.202501140)
Supplement: Supplementary file 1 — Supporting Information [file ADVS-12-e01140-s001.docx]

Supporting Information

**Releasing 8.0 wt.% H_2_ from the LiBH_4_−2LiNH_2_ Composite within 5 Min under Light Illumination**

Haoyang Yu, Zibo Cheng, Hong Wen, Han Wang, Qijun Pei, Yeqin Guan, Hujun Cao* and Ping Chen

**Supplementary Figures**


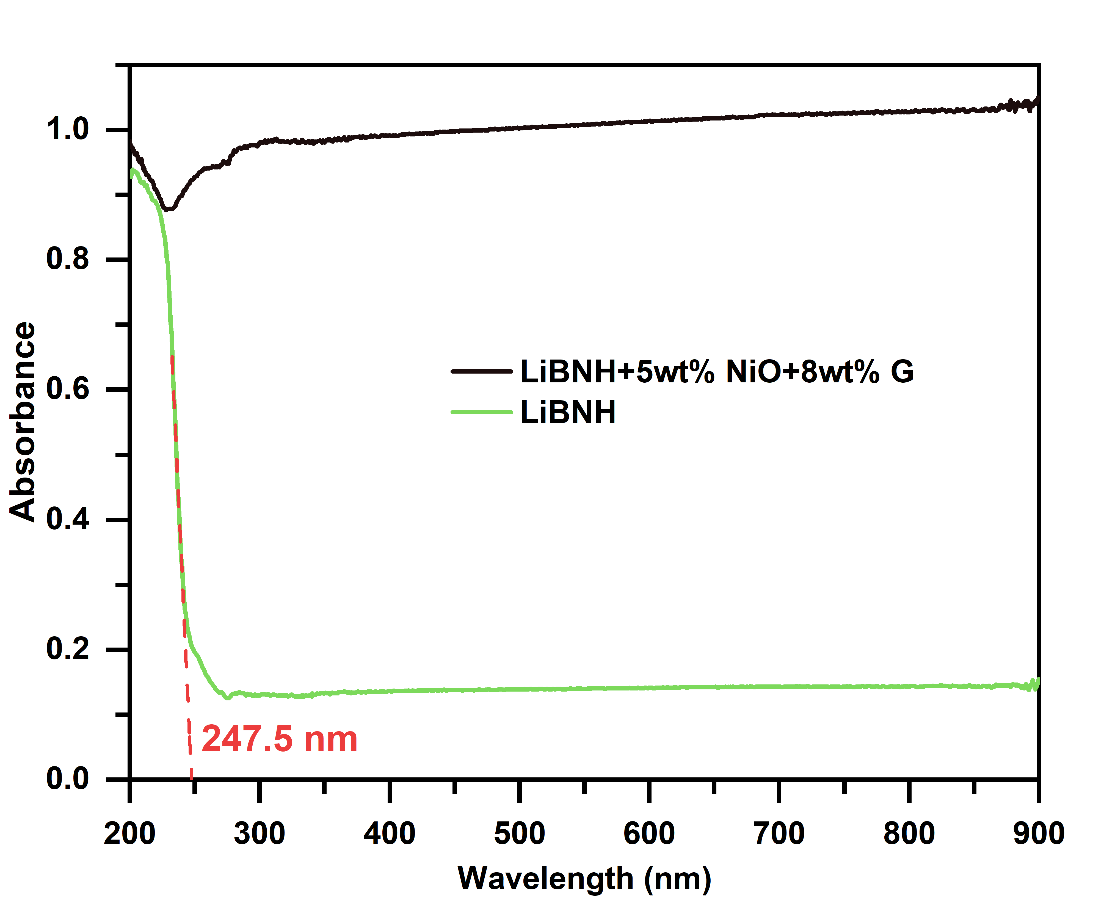


**Figure S1** UV–vis absorption spectrum of LiBNH with/without the addition of nickel oxide and graphene.

**Figure S2** Real-time infrared images of LiBNH+5wt.%NiO+8wt.%G under the full spectrum of 1.94 W·cm^−2^.


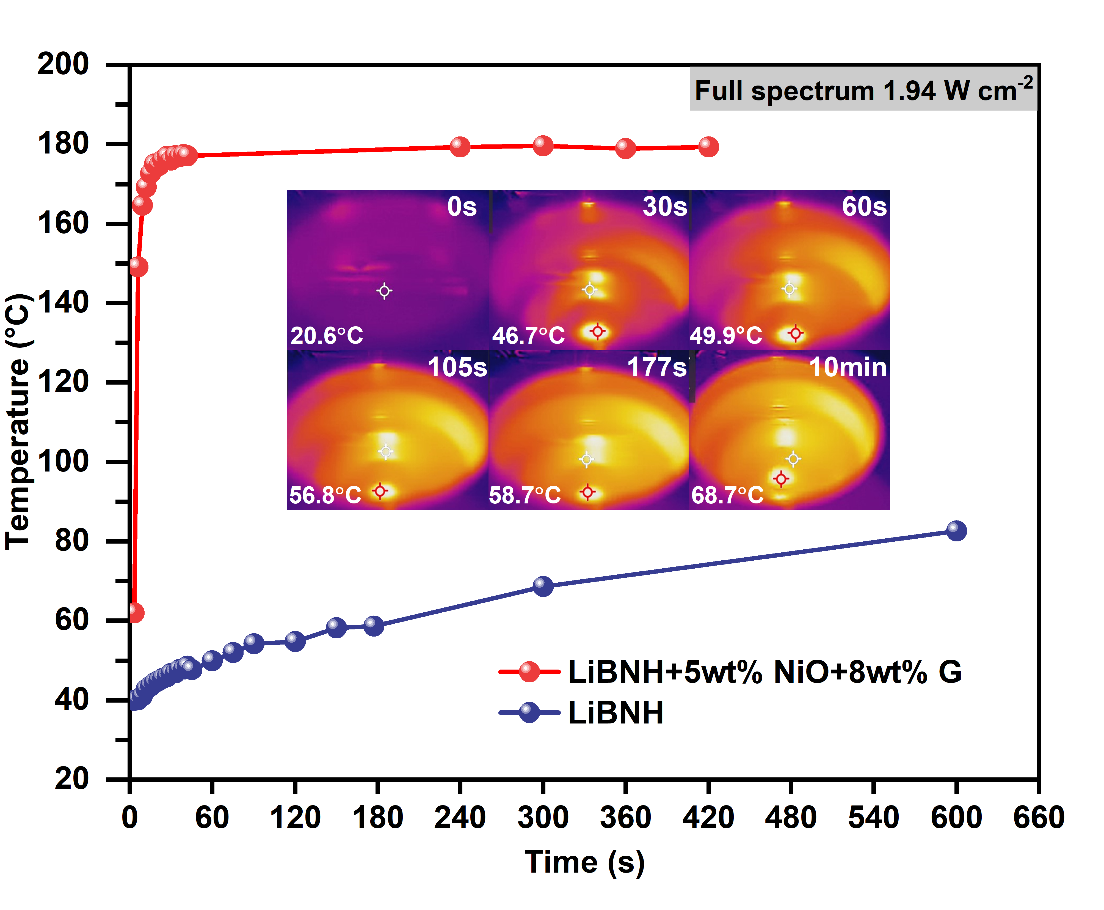


**Figure S3** Real-time infrared temperature curve under the full spectrum of 1.94 W·cm**^−^**^2^. Inset is the infrared image.


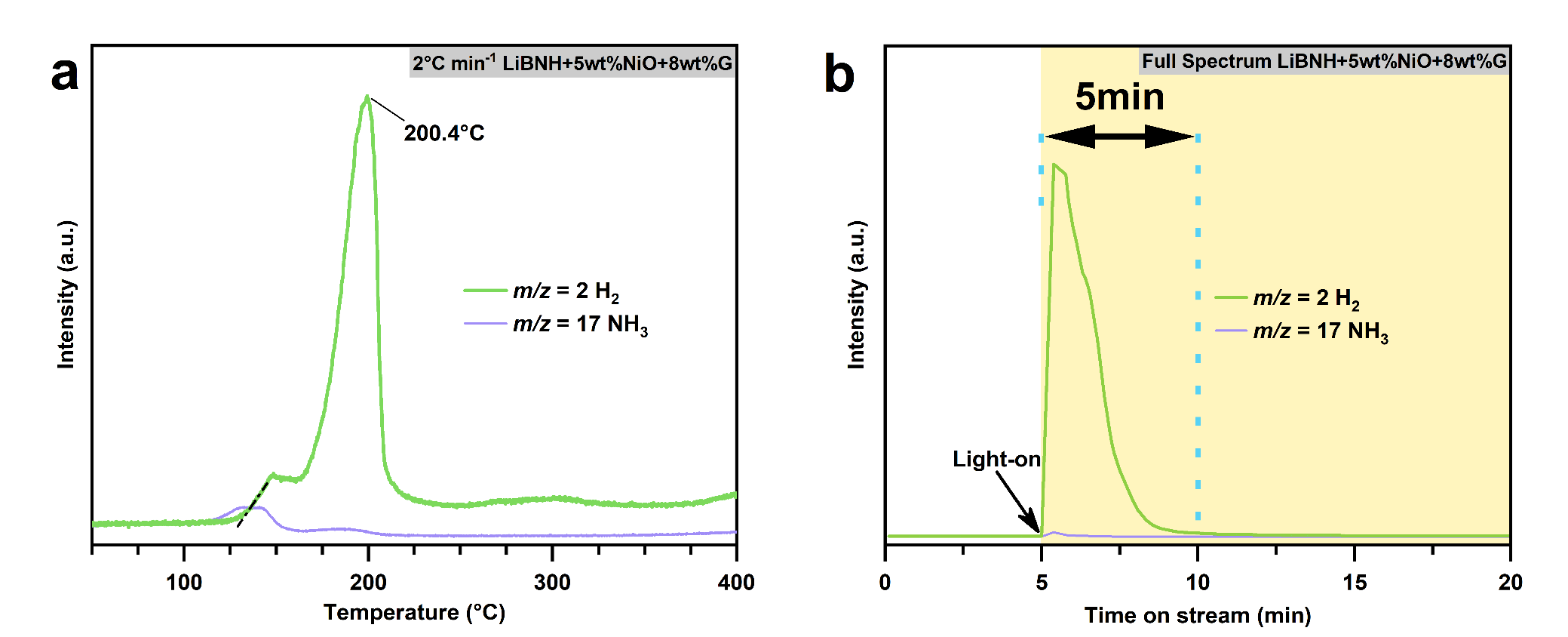


**Figure S4** a) Temperature programmed thermal-driven and b) photo-driven dehydrogenation of LiBNH+5wt.%NiO+8wt.%G in the mass spectrometry.


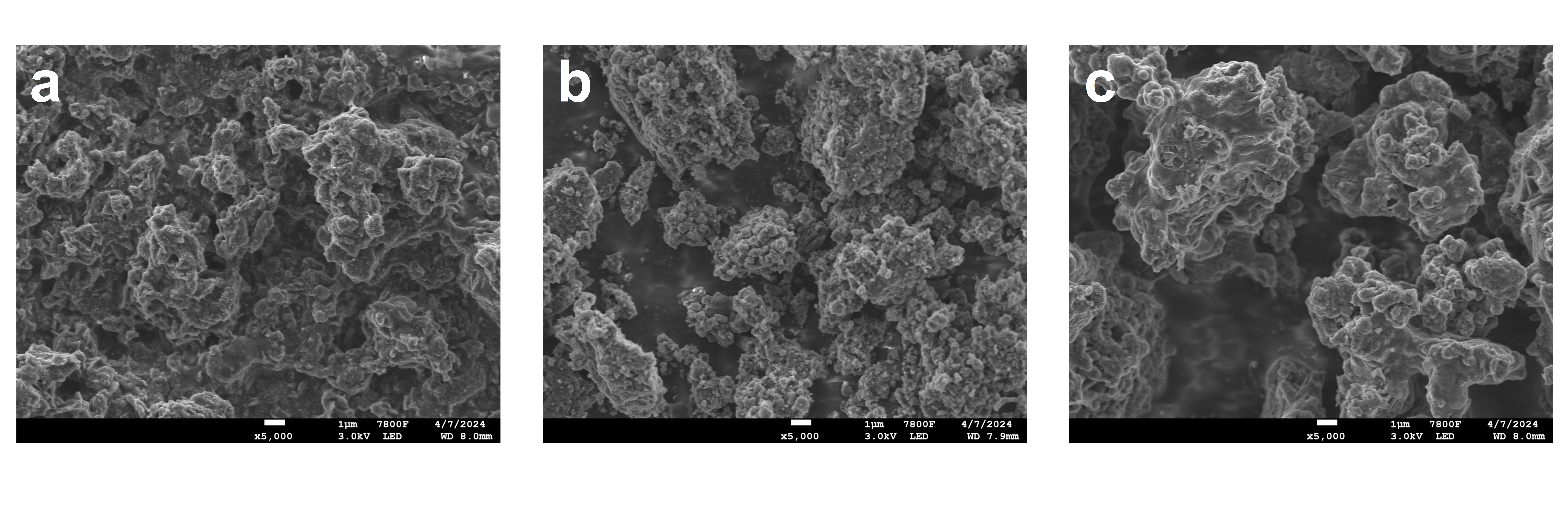


**Figure S5** SEM images of LiBNH+5wt.%NiO+8wt.%G a) as-prepared, b) photo-driven dehydrogenated, and c) thermal-driven dehydrogenated.


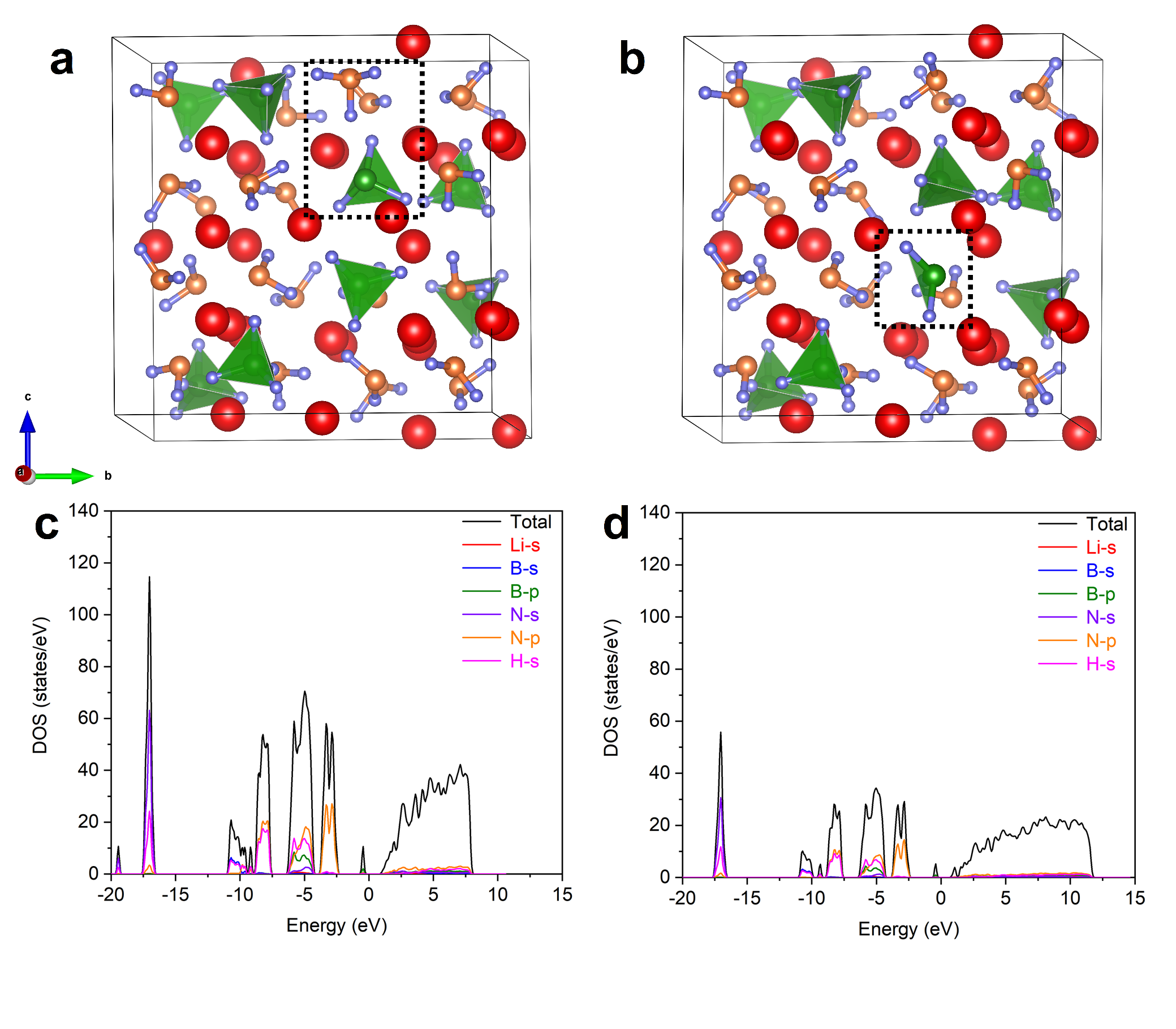


**Figure S6** The optimized Li_4_BN_3_H_10_ crystal structure with an ammonia molecule as an intermediate state a), and the crystal structure after ammonia molecule release b), along with their density of states (DOS) c) and d), respectively.


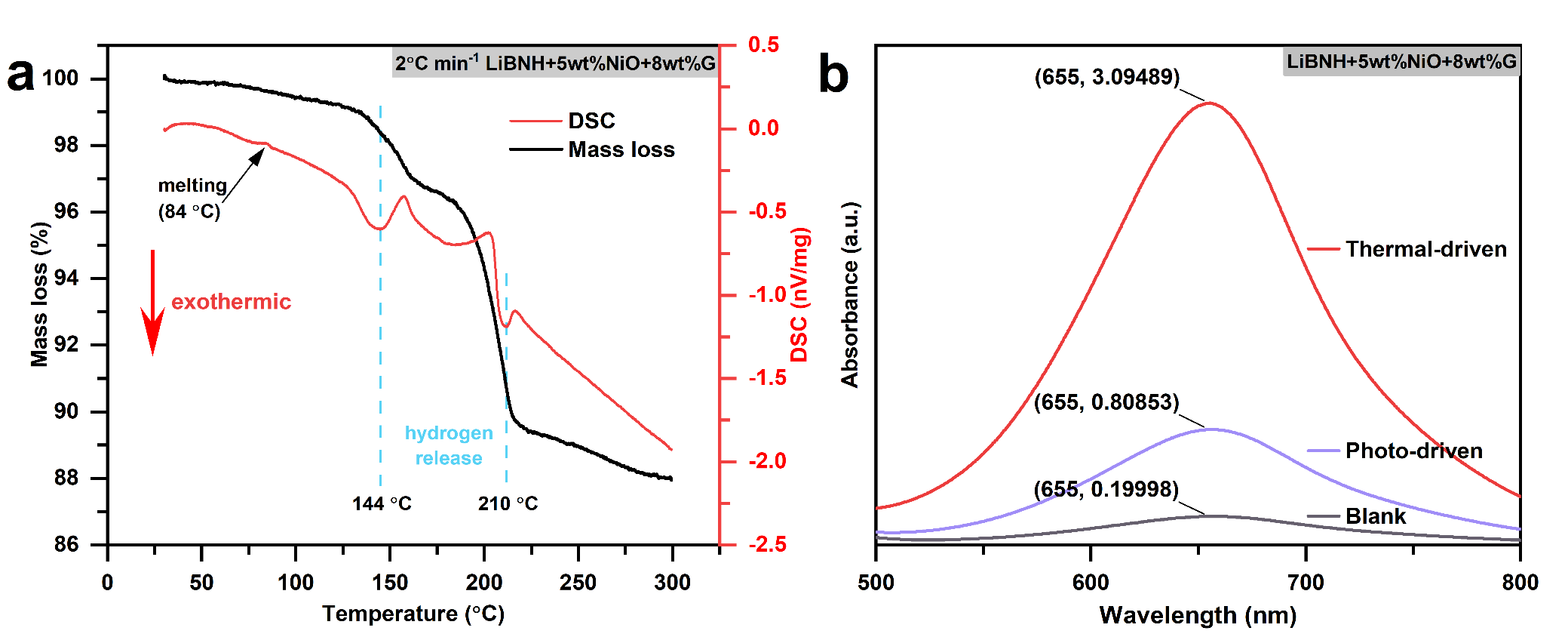


**Figure S7** a) TG−DSC curve of LiBNH+5wt.%NiO+8wt.%G in the temperature range of 30 to 300 °C, with a heating rate of 2 °C·min^−1^, b) Spectrophotometric determination of ammonia release driven by photo and thermal.

The TG–DSC device is used to measure the released gaseous (Figure S7a). Ammonia gas is quantified using the UV–vis absorption method (Figure S7b). Interestingly, the amount of deamination driven by light is significantly lower than that driven by heat, accounting for only one-fifth. With the assistance of TG, it is found that the specific gas ammonia concentration decreases from ca.2.46 mol% to 0.52 mol%. The decreased ammonia concentration can be attributed to the distinction between these two dehydrogenation drive forces, specifically the non-thermal effects associated with the photo-driven processes. This finding highlights the benefits of ultraviolet irradiation on the dehydrogenation process in the LiBNH system.

The calculation process is as follows:

Assume the released gases contain x mol H_2_ and y mol NH_3_. Based on the weight loss data from TG analysis and the hydrogen atom count in the LiBH_4_−2LiNH_2_ molecule, a set of equations can be established according to the principles of mass conservation (Equation S1) and hydrogen atom conservation (Equation S2).

2x+17y=(m×12%)/1000 Equation (S1)

2x+3y=(8×m×87%)/(67.712×1000) Equation (S2)

x=4.955×m×10^−5^; y=1.229×m×10^−6^ Equation (S3)

y/x≈2.46 mol% Equation (S4)

**Figure S8** The optimized crystal structure of Li_2_BNH_6_ is shown in a) a ball-stick view containing [BH_4_]^−^ tetrahedral units (in green and violet) and [NH_2_]^−^ units (in orange and violet), with Li^+^ ions represented as red spheres. b) Crystal structure of Li_2_BNH_6_ after desorption of one H_2_ molecule. c) Density of states (DOS) for the Li_2_BNH_6_. d) Density of states (DOS) for the Li_2_BNH_6_ after the removal of one H_2_ molecule.

**Figure S9** The optimized Li_2_BNH_6_ crystal structure with an ammonia molecule as an intermediate state a), and the crystal structure after one ammonia molecule release b), and their density of states (DOS) c) and d) respectively.


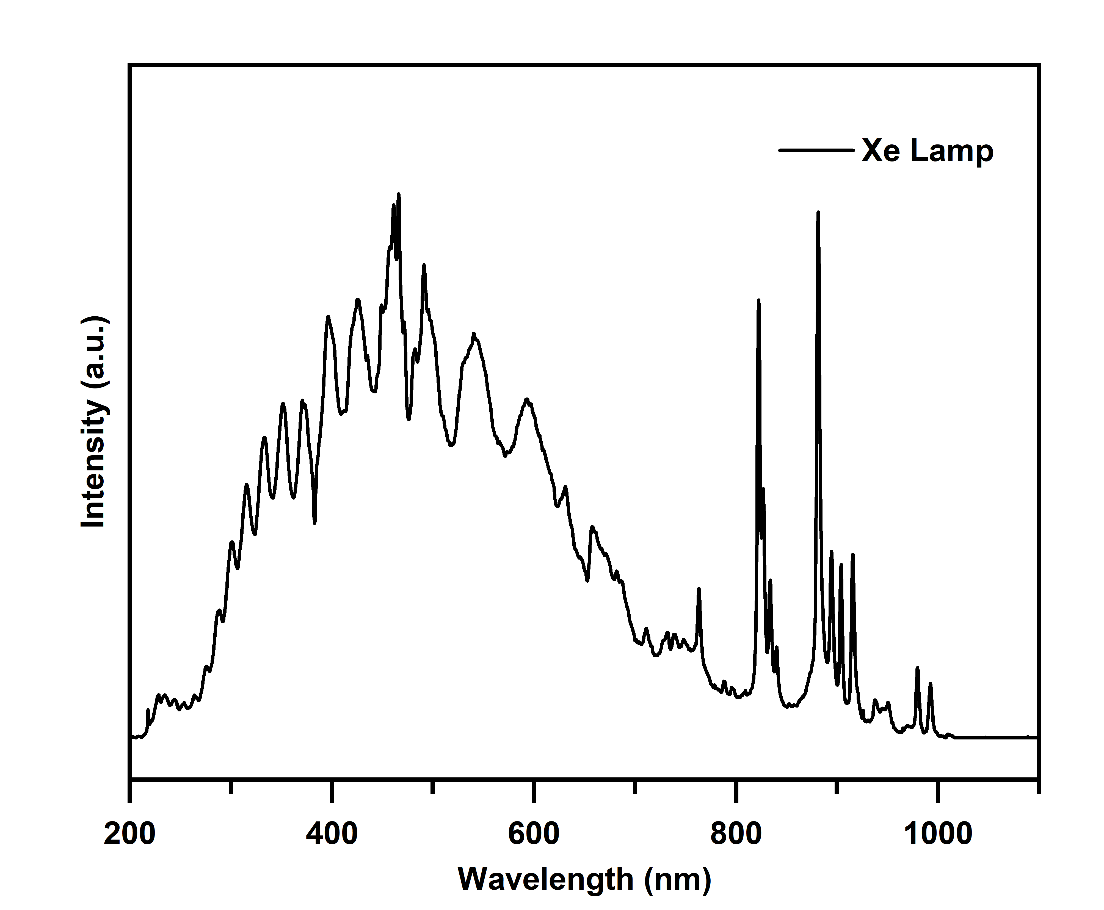


**Figure S10** Light spectra of the full-spectrum Xe Lamp.

**Table S1** Theoretical calculation of the energy of the optimized crystal structure

| structure | E_gap_/eV | [NH_2_]−H(BH_4_) | | |
| --- | --- | --- | --- | --- |
|  |  | H_2_ E_form_/ eV | (NH_3_)_i_ E_form_/ eV | (NH_3_)_g_ E_form_/ eV |
| Li_2_BNH_6_ | 4.22 | 0.97 | 2.67 | 3.78 |
| Li_4_BN_3_H_10_ | 3.69 | 1.24 | 2.71 | 3.51 |

(NH_3_)_i_: Absorbed ammonia in the intermediate state.

(NH_3_)_g_: Released free ammonia in gas state.

**Table S2** Bond changes after ammonia molecule desorption

| structure | group | bond | [NH_2_]−H(BH_4_) | (NH_3_)_i_  [NH_2_]−H(BH_4_) | (NH_3_)_g_  [NH_2_]−H(BH_4_) |
| --- | --- | --- | --- | --- | --- |
| Li_2_BNH_6_ | NH_2_ | N−H_1_ | 1.03 | 1.03 | 1.03 |
|  |  | N−H_2_ | 1.03 | 1.03 | / |
|  |  | N−H_3_ | / | 1.08 | / |
|  | BH_4_ | B−H_1_ | 1.23 | / | / |
|  |  | B−H_2_ | 1.23 | 1.24 | 1.24 |
|  |  | B−H_3_ | 1.23 | 1.24 | 1.24 |
|  |  | B−H_4_ | 1.23 | 1.24 | 1.23 |
| Li_4_BN_3_H_10_ | NH_2_ | N−H_1_ | 1.03 | 1.03 | 1.03 |
|  |  | N−H_2_ | 1.03 | 1.03 | / |
|  |  | N−H_3_ | / | 1.07 | / |
|  | BH_4_ | B−H_1_ | 1.23 | / | / |
|  |  | B−H_2_ | 1.23 | 1.24 | 1.24 |
|  |  | B−H_3_ | 1.23 | 1.24 | 1.23 |
|  |  | B−H_4_ | 1.23 | 1.25 | 1.23 |

(NH_3_)_i_: Absorbed ammonia in the intermediate state.

(NH_3_)_g_: Released free ammonia in gas state.

All units are in Å

**Table S3** Bond length after hydrogen molecule desorption

| structure | bond | H_2_ [NH_2_]−H(BH_4_) |
| --- | --- | --- |
| Li_2_BNH_6_ | Li−B | 2.23 |
|  | Li−N | 2.03 |
|  | N−B | 1.55 |
|  | N−H | 1.027 |
|  | B−H_1_ | 1.24 |
|  | B−H_2_ | 1.26 |
|  | B−H_3_ | 1.27 |
| Li_4_BN_3_H_10_ | Li−B | 2.14 |
|  | Li−N | 2.01 |
|  | N−B | 1.56 |
|  | N−H | 1.025 |
|  | B−H_1_ | 1.23 |
|  | B−H_2_ | 1.26 |
|  | B−H_3_ | 1.27 |

All units are in Å
